# Cognitive map formation in the blind is enhanced by three-dimensional tactile information

**DOI:** 10.1038/s41598-023-36578-3

**Published:** 2023-06-15

**Authors:** Maxime Bleau, Camille van Acker, Natalina Martiniello, Joseph Paul Nemargut, Maurice Ptito

**Affiliations:** 1 School of Optometry, University of Montreal, Montreal, QC Canada; 2 Institut Royal Pour Sourds et Aveugles, Brussels, Belgium; 3 Department of Neuroscience, University of Copenhagen, Copenhagen, Denmark; 4 Department of Neurology and Neurosurgery, Montreal Neurological Institute, McGill University, Montreal, QC Canada

**Keywords:** Psychology, Spatial memory, Perception, Cognitive neuroscience, Sensory processing

## Abstract

For blind individuals, tactile maps are useful tools to form cognitive maps through touch. However, they still experience challenges in cognitive map formation and independent navigation. Three-dimensional (3D) tactile information is thus increasingly being considered to convey enriched spatial information, but it remains unclear if it can facilitate *cognitive map formation* compared to traditional two-dimensional (2D) tactile information. Consequently, the present study investigated the impact of the type of sensory input (tactile 2D vs. tactile 3D vs. a visual control condition) on *cognitive map formation*. To do so, early blind (EB, n = 13), late blind (LB, n = 12), and sighted control (SC, n = 14) participants were tasked to learn the layouts of mazes produced with different sensory information (tactile 2D vs. tactile 3D vs. visual control) and to infer routes from memory. Results show that EB manifested stronger cognitive map formation with 3D mazes, LB performed equally well with 2D and 3D tactile mazes, and SC manifested equivalent cognitive map formation with visual and 3D tactile mazes but were negatively impacted by 2D tactile mazes. 3D tactile maps therefore have the potential to improve spatial learning for EB and newly blind individuals through a reduction of cognitive overload. Installation of 3D tactile maps in public spaces should be considered to promote universal accessibility and reduce blind individuals’ wayfinding deficits related to the inaccessibility of spatial information through non-visual means.

## Introduction

Orientation and independent spatial navigation are intricate behaviors that depend on highly complex cognitive processes^1^. Orientation is defined as: “The knowledge of one’s distance and direction relative to things observed or remembered in one’s surroundings and the ability to keep track of these spatial relationships as they change during locomotion”^2–5^. While most humans automatically and effortlessly use visual information to establish and maintain orientation^6^, those living with severe visual impairments or blindness must learn to rely on less precise sensory channels, such as touch (proximal), audition (distal), olfaction (distal), and proprioception (internal), to gather and encode spatial information and navigate^7,8^. To date, orientation, or *wayfinding*, is still one of the main daily challenges of visually impaired individuals, which can lead to decreased autonomy and confidence^9^, increased anxiety before and during navigation, disorientation, and ultimately to decreased professional opportunities and societal isolation^10,11^.

In fact, orientation in the absence of vision is heavily dependent on individuals’ ability to form *cognitive maps* of their surroundings^2^. A cognitive map is defined as an internal mental representation of an environment that preserves spatial properties (i.e., locations) and relationships (i.e., distances and directions) between memorized landmarks, paths, or other environmental features^2,3,12,13^. Such cognitive maps are therefore allocentric, or viewpoint-independent, and flexible in a way that enables individuals to infer routes and detours despite barriers and regardless of their location and viewpoint^14^. Cognitive map formation, or spatial learning, is thus an essential skill for navigation as it enables better route planning and improves problem solving skills. For example, individuals with good cognitive maps can more easily realize if they stray away from a planned route and then correct themselves instead of becoming disoriented. Although blind individuals can form such cognitive maps and gain efficient spatial knowledge to guide navigation, spatial learning is a heavily serialized process that requires significant exposure to environmental features, and thus more time and cognitive effort for memorization^15,16^. It is therefore likely for blind individuals to be disoriented in complex spaces that require a lot of turns and non-linear movements^17^.

Tactile maps are effective tools used by blind individuals to facilitate cognitive map formation in novel small- and large-scale environments. As a tactile substitution for visual maps, they only provide essential spatial information and preserve all spatial relationships between landmarks, destinations, or objects in the environment^1^. These can be rapidly explored by the user, thus providing a relatively simultaneous aerial view of an environment while sidelining many difficulties linked with real-time exploration such as various dangers, anxiety, veering, disorientation, and the constant strain on memory^18–20^. Tactile maps can thus provide a survey, or allocentric, understanding of an environment in a more effective way than direct experience^18,21,22^. Because of their usefulness, there are now numerous technologies and ways to produce tactile maps^23^. However, early blind (EB) still experience challenges in orientation and tactile map reading^6,24–27^.

In recent years, advances in 3D printing technology have spurred interest in the field of vision rehabilitation^28^ and have led many researchers to investigate the potential of 3D printers to automatically produce tactile maps^29–32^. Indeed, 3D scale models like those produced with 3D printing are viewed as beneficial and durable tools to convey spatial information and facilitate orientation in indoor and outdoor spaces^33–35^. Gual-Orti and colleagues^36–39^ showed that volumetric symbols produced by 3D printing can be understood, easily memorized, and used to efficiently follow routes. Holloway and colleagues^40,41^ demonstrated that completely 3D printed tactile maps, when compared to traditional 2D embossed tactile graphics, were advantageous for the memorization of information and for the comprehensibility of harder concepts such as depth and occlusion (i.e., a bridge over a pathway). They also showed that blind individuals preferred these models over their 2D counterparts^41^. However, there is less evidence on how, and if, 3D tactile information can improve cognitive map formation at a functional level.

Therefore, the present study investigated, for the first time with quantitative measures, the impact of tactile 3D information on the ability of blind and sighted subjects to explore mazes and form functional allocentric cognitive maps. To do so, we compared equivalent 2D swell paper and 3D printed tactile maps in an original paradigm of “contextual mazes”. Furthermore, since age at onset of blindness and visual experience have a significant impact on brain development and performance in complex spatial tasks^6^, we tested subjects with early- and late-onset blindness as well as sighted controls who also performed the task visually. We hypothesized that (1) mazes that carry more information in the 3rd dimension (vertically) will lead to more accurate cognitive maps than 2D mazes for all groups; and (2) the 3rd dimension would be particularly beneficial to those lacking visual experience, but that vision will still lead to better cognitive maps in the sighted.

## Results

### Symbol localization (detection + identification) time

**Figure 1:**
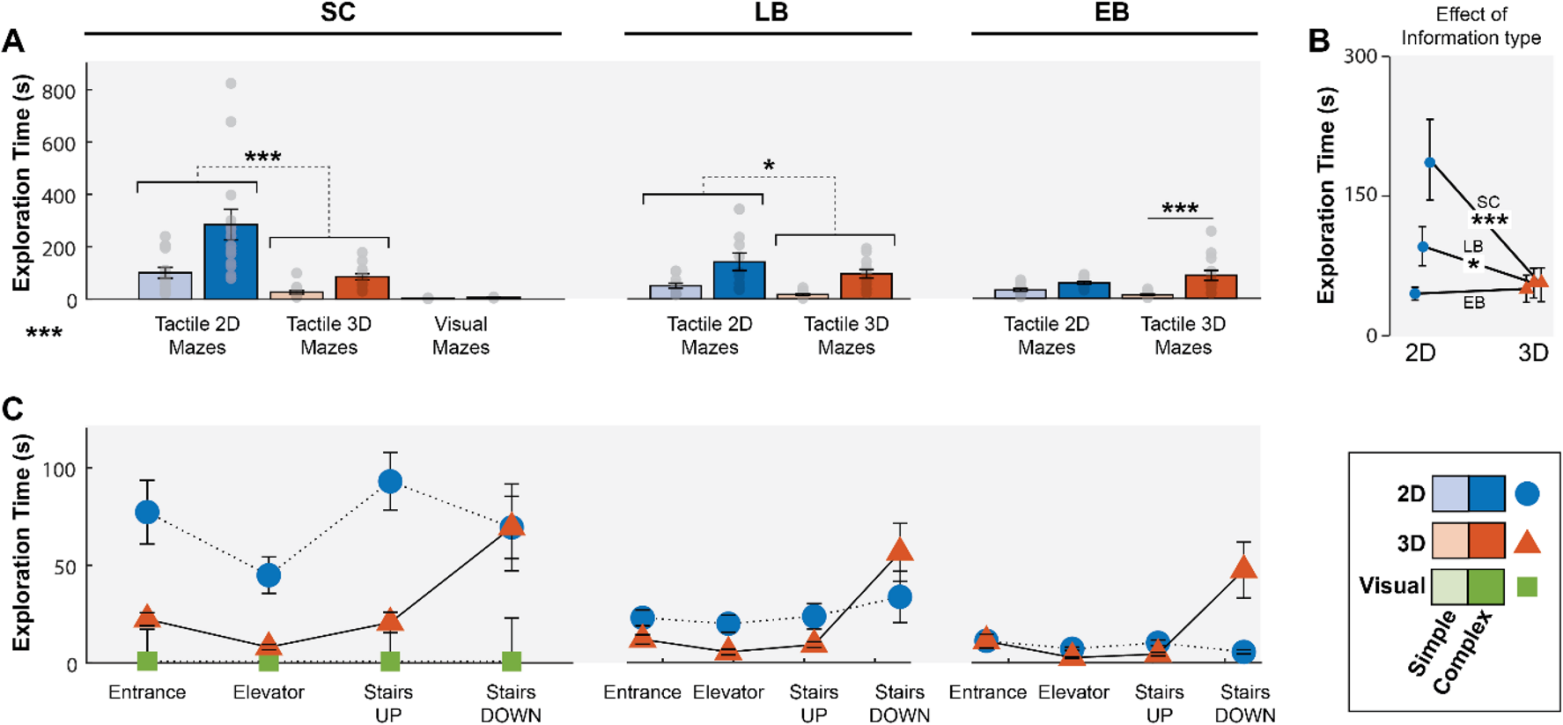
Symbol localization times for the three information types (tactile 2D, tactile 3D, and visual). Symbol localization times combine detection and identification of symbols. (**A**) Symbol localization times for simple and complex mazes. Symbol localization times in 2D mazes were significantly longer than in 3D mazes for both SC (***) and LB (*); and were longer in complex 3D mazes than in simple 3D mazes for EB (***). (**B**) Effect of information type for all three groups shows a higher variability between groups in 2D mazes (SC being the slowest) and the equivalence of all groups in 3D mazes. (**C**) Symbol localization times according to each symbol type. SC symbol localization times for 2D symbols were significantly longer than any other symbol localization times. The error bars illustrate the standard error of the mean. EB, early blind; LB, late blind; SC, sighted control; 2D, two-dimensional; 3D, three-dimensional; *, *p* < 0.05; ***, *p* < 0.001.

As for symbol localization times (symbol detection and identification), SC took 92.25 ± 73.87 s to find all symbols in simple 2D mazes, 284.74 ± 226.71 s in complex 2D mazes, 24.31 ± 24.66 s in simple 3D mazes, 85.07 ± 42.87 s in complex 3D mazes, 2.49 ± 0.90 s in simple visual mazes, and 5.84 ± 1.28 s in complex visual mazes. LB took 50.46 ± 31.77 s in simple 2D mazes, 141.63 ± 115.66 s in complex 2D mazes, 16.75 ± 12.08 s in simple 3D mazes, and 96.08 ± 55.51 s in complex 3D mazes. Finally, EB took 31.85 ± 19.15 s in simple 2D mazes, 58.19 ± 19.05 s in complex 2D mazes, 13.12 ± 9.05 s in simple 3D mazes, and 87.39 ± 69.00 s in complex 3D mazes. These results are illustrated in Fig. 1A.

When looking at SC in all conditions (including the visual control condition), it is noteworthy that SC almost immediately detected visual symbols but took longer for tactile symbols, and more so for 2D than 3D symbols. However, when performing the 2 × 3 ANCOVA (corrected for age and sex), this effect of information type was cancelled out by the effect of age. Indeed, the test revealed a significant interaction between age and information type (F(1.06, 10.64) = 18.022, *p* < 0.001***, ${\eta }_{p}^{2}$ = 0.643), while a three-way interaction between age, information type and complexity tended towards significance (F(1.064,10.638) = 3.761, *p* = 0.078). This indicate that age is an important factor in SC symbol localization times.

When analyzing symbol localization times for the three groups in the tactile 2D and 3D conditions, the 3 × 2 × 2 ANCOVA (corrected for age and sex) revealed three-way interactions between groups, information type and complexity (F(2,33) = 4.721, *p* < 0.05*, ${\eta }_{p}^{2}$ = 0.222), and between participants’ age, information type and complexity (F(1,33) = 7.496, *p* < 0.05*, ${\eta }_{p}^{2}$ = 0.185). To investigate the first three-way interaction (group*information type*complexity), 2 × 2 ANOVAs were performed to investigate the two-way interaction between information type and complexity for the three groups separately. This interaction was only significant in EB (F(1,12) = 28.699, *p* < 0.001***, ${\eta }_{p}^{2}$ = 0.358). Simple main effects revealed that EB symbol localization times were impacted by complexity for both 2D (F(1,12) = 19.683, *p* < 0.001***) and 3D mazes (F(1,12) = 17.657, *p* < 0.001***), but post-hoc T-tests with Bonferroni correction revealed that EB took less time to find symbols in simple mazes only in the 3D tactile condition (mean difference = − 74.269 s, t = − 5.633, *p* < 0.001***). This indicates that EB were so efficient in exploring 2D mazes that the number of symbols did not affect their symbol localization time while it did for 3D mazes (with which they were previously unfamiliar, see Fig. 1B). This is accentuated by the fact that EB were not affected by information type (F(1,12) = 0.263, *p* = 0.617), meaning that, despite the effect of complexity in 3D mazes, they were equally fast in 2D than in 3D. On the contrary for LB and SC, ANOVAs revealed that both were impacted by information type (LB: F(1,11) = 6.313, *p* < 0.05*, ${\eta }_{p}^{2}$ = 0.365; SC: F(1,12) = 19.271, *p* < 0.001***, ${\eta }_{p}^{2}$ = 0.616) and complexity (LB: F(1,11) = 23.011, *p* < 0.001***, ${\eta }_{p}^{2}$ = 0.677; SC: F(1,12) = 17.445, *p* < 0.01**, ${\eta }_{p}^{2}$ = 0.592). Post-hoc T-tests with Bonferroni correction revealed that both groups took longer to find symbols in complex mazes (LB: mean difference = 85.25 s, t = 4.797, *p* < 0.001***; SC: mean difference = 126.627 s, t = 4.177, *p* < 0.01**) and were faster to find symbols in 3D when compared to 2D (LB: mean difference = -39.625 s, t = 2.513, *p* < 0.05*; SC: mean difference = − 133.804 s, t = -4.390, *p* < 0.001***). This last result is illustrated in Fig. 1B. It is also interesting to note that the interaction between information type and complexity was almost significant in SC (F(1,12) = 4.243, *p* = 0.062, ${\eta }_{p}^{2}$ = 0.261). This might be due to SC symbol localization times for 2D mazes being longer than all other recorded symbol localization times (see Fig. 1) and to the effect of age on SC tactile exploration of 2D mazes.

When taking symbol identities into account, the 3 × 2 × 4 ANCOVA (corrected for age and sex) revealed two significant two-way interaction effects. The first interaction was between information type and group (F(1,32) = 13.289, *p* < 0.001***, ${\eta }_{p}^{2}$ = 0.454): a test for simple main effects revealed that only SC was affected by information type (F(1,13) = 9.401, *p* < 0.05*), an effect that did not survive the correction for the age covariate (F(1,12) = 0.043,* p* = 0.840). This indicates that age is an important factor in SC symbol localization times. Nonetheless, we note that SC took longer times finding 2D symbols when compared to 3D symbols (see Fig. 1). The second interaction was between information type and age (F(1,33) = 6.666, *p* < 0.05*, ${\eta }_{p}^{2}$ = 0.172). Pearson’s r tests were performed to investigate the correlation between age and symbol localization times for 2D and 3D tactile conditions separately. These analyses are presented in the “Effects of age and SBSOD score on symbol localization time and route performance” section. Finally, the ANCOVA also revealed an additional effect of symbol identity (F(1.82,58.31) = 3.418, *p* < 0.05*, ${\eta }_{p}^{2}$ = 0.096) on symbol localization times. Indeed, only the “descending staircase” took significantly longer to find than any other symbols (*p* < 0.01**), an effect likely caused by the time taken by all groups to find the 3D version of the symbol (see Fig. 1C).

### POI recall performance

Q-Q plots revealed deviations from normality in the distributions of points of interest (POI) recall performances. Therefore, a GLMM analysis was conducted to examine the effects of information type and complexity on POI recall performance, taking into account the grouping factor as a random effect in the model. Contrary to our hypothesis, the analysis revealed only a significant effect of complexity (β = − 0.083, SE = 0.0280, t = − 2.961, *p* = 0.004, 95% CI [− 0.138, − 0.028]), but no significant effect of information type (β = − 0.036, SE = 0.0280, t = − 1.274, *p* = 0.205, 95% CI [− 0.091, 0.020]). Furthermore, the grouping factor accounted for a negligible proportion of the variance. These results demonstrate that the POI recall performance did not change across groups and was only affected by complexity. Indeed, average POI recall performance was 95.73% for simple mazes and decreased to 85.73% for complex mazes.

### Route elaboration performance

**Figure 2:**
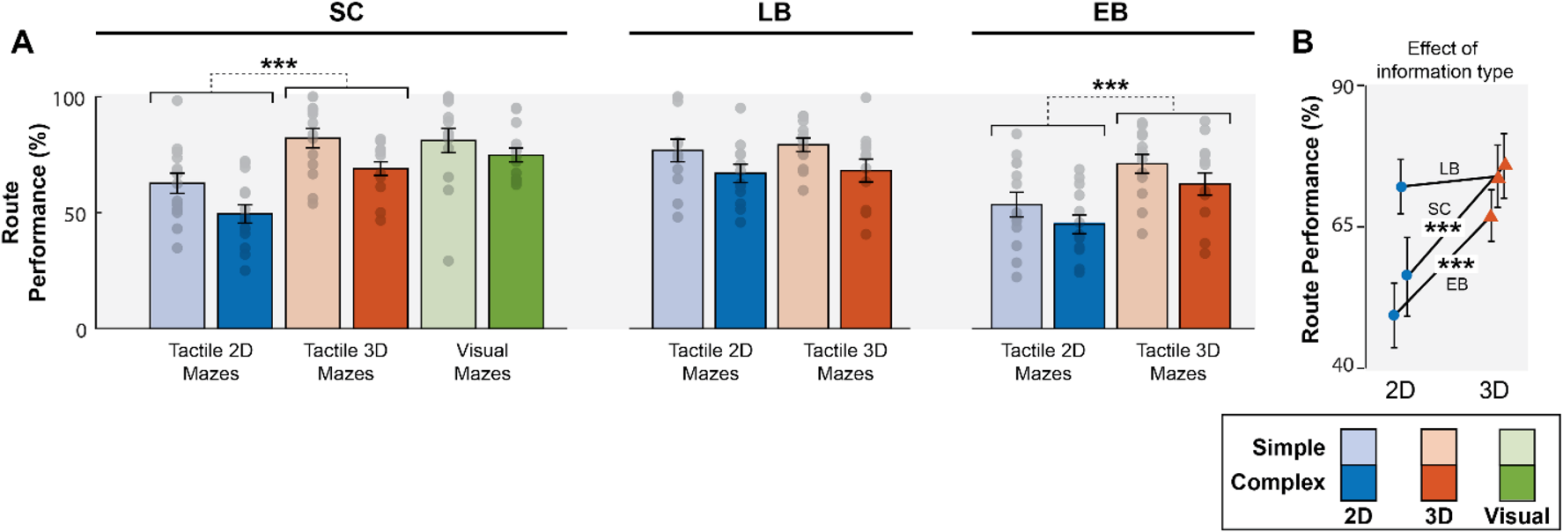
Route elaboration performance. (**A**) Route performance (in %) was higher in 3D mazes than 2D mazes for SC (***) and EB (***), but not LB. (**B**) The effect of information type for all three group shows that all groups are equivalent in tactile 3D mazes, but not in tactile 2D mazes for which EB and SC performance decreased. The error bars illustrate the SEM. EB, early blind; LB, late blind; SC, sighted control; 2D, two-dimensional; 3D, three-dimensional; ***, *p* < 0.001.

As for route elaboration performance, or the measure of cognitive mapping skills, SC obtained average performances of 62.62 ± 16.53% in simple 2D mazes, 49.46 ± 15.08% in complex 2D mazes, 82.10 ± 15.65% in simple 3D mazes, 68.95 ± 10.98% in complex 3D mazes, 81.08 ± 19.84% in simple visual mazes, and 74.84 ± 11.53% in complex visual mazes. LB obtained 76.81 ± 17.06% in simple 2D mazes, 66.96 ± 13.79% in complex 2D mazes, 79.26 ± 10.43% in simple 3D mazes, and 68.95 ± 10.98% in complex 3D mazes. EB obtained 53.54 ± 19.54% in simple 2D mazes, 45.01 ± 14.41% in complex 2D mazes, 71.14 ± 14.75% in simple 3D mazes, and 62.44.01 ± 17.51% in complex 3D mazes. These results are illustrated in Fig. 2A.

When looking at SC route performance in all conditions (including the visual control condition), we notice that SC performed equally well in the tactile 3D and the visual control conditions, while their performance decreased in the tactile 2D condition. However, when performing the 2 × 3 ANCOVA, this effect of information type was cancelled out by the effect of the age covariate (F(1,10) = 3.999, *p* = 0.073, trend). The test thus only revealed a significant effect of maze complexity (F(1,11) = 5.116, *p* < 0.05*, ${\eta }_{p}^{2}$ = 0.317). Indeed, SC performed better in simple mazes (mean difference = 12.198%, t = 4.85, *p* < 0.001***).

When analyzing route performance for the three groups in the tactile 2D and 3D conditions, the 3 × 2 × 2 ANCOVA (corrected for age and sex) revealed a significant maze complexity effect (F(1,34) = 9.263, *p* < 0.01**, ${\eta }_{p}^{2}$ = 0.214) and a significant interaction between information type and group (F(2,34) = 15.411, *p* < 0.001***, ${\eta }_{p}^{2}$ = 0.475). This interaction indicates that the three groups performed differently in relation to information type. Indeed, simple main effects revealed that information type affected both EB (F(1,12) = 33.722, *p* < 0.001***) and SC (F(1,13) = 36.994, *p* < 0.001***), but not LB (F(1,11) = 0.371, *p* = 0.558). However, when correcting for the age covariate, the main effect of information type on EB remained (F(1,11) = 6.904, *p* < 0.05*), but disappeared for SC (F(1,12) = 1.714, *p* = 0.217). Since it was noticed that age negatively impacted SC performance in route elaboration (see next section), we further investigated the effect of information type on route elaboration performance for SC who were older than 50 years old (n = 7) compared to those who were younger than 50 years old (n = 7) with a 2 × 2 ANOVA. The test revealed no interaction between the age group and information type (F(1,12) = 0.419, *p* = 0.530), but a significant effect of information type (F(1,12) = 61.554, *p* < 0.001***). Post-hoc T-tests with Bonferroni correction revealed that 3D mazes improve performance for both SC (t = 7.846, *p* < 0.001***) and EB (t = 6.929, *p* < 0.001***), but not LB (t = 0.455, *p* = 1). This reveals that 3D tactile information can improve the cognitive mapping skills of individuals who lack either tactile experience (SC) or visual experience (EB), but not those who cumulate both (LB). All route elaboration results are illustrated in Fig. 2.

### Effects of age and SBSOD score on symbol localization time and route performance

**Figure 3:**
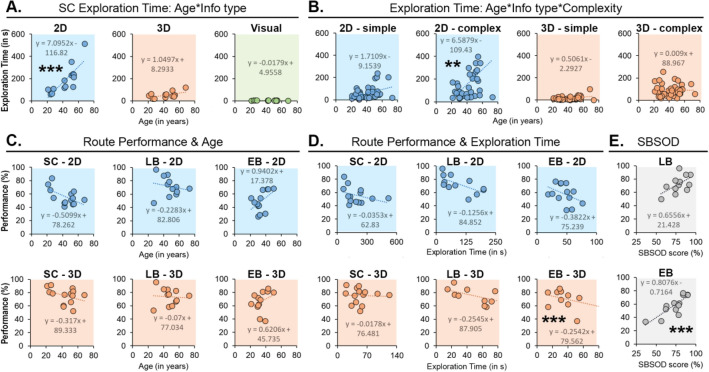
Pearson’s R tests results for age, symbol localization time, route performance, and SBSOD scores. (**A**) Relation between age and symbol localization time for SC. These are positively correlated in 2D mazes (***). (**B**) Relation between age and symbol localization time in simple and complex mazes (all groups combined). These are positively correlated in 2D complex mazes (**). (**C**) Relation between age and route performance. These appear negatively related, except for EB. (**D**) Relation between route performance and symbol localization time. These appear negatively related (only significant for 3D mazes in EB, ***). (**E**) Relation between route performance and SBSOD scores. These are positively related (significant for EB, ***). Trend lines and their equations are illustrated for every individual test. EB, early blind; LB, late blind; SBSOD, Santa Barbara Sense of Direction; SC, sighted control; 2D, two-dimensional; 3D, three-dimensional; **, *p* < 0.01; ***, *p* < 0.001.

Pearson’s R tests were performed to investigate the previously identified effects of age on symbol localization time and route elaboration performance. For SC, age correlated with symbol localization time in 2D mazes (Pearson’s r = 0.807, *p* < 0.001***, ${\mathrm{p}}_{\mathrm{bonf}}$<0.001***). This indicates that symbol localization of 2D mazes becomes less efficient with age for SC, which was true for simple (pearson’s r = 0.599, *p* = 0.024*, ${\mathrm{p}}_{\mathrm{bonf}}$ = 0.048*) and complex (pearson’s r = 0.701, *p* = 0.005**, ${\mathrm{p}}_{\mathrm{bonf}}$ = 0.01*) mazes. This stronger relationship in 2D complex mazes may explain why SC had slower recorded times in this condition (see Fig. 3A). Then, for EB, LB, and SC combined, we looked at the three-way interaction effect (age*information type*complexity) on symbol localization time by assessing the correlation between age and symbol localization times for all tactile conditions. A correlation was found in complex 2D mazes (Pearson’s r = 0.493, *p* = 0.001**, ${\mathrm{p}}_{\mathrm{bonf}}$ = 0.004*), while there was a trend towards significance in simple 2D mazes (Pearson’s r = 0.381, *p* = 0.017*, ${\mathrm{p}}_{\mathrm{bonf}}$ = 0.068) and simple 3D mazes (Pearson’s r = 0.375, *p* = 0.019*, ${\mathrm{p}}_{\mathrm{bonf}}$ = 0.076). This indicates that, regardless of tactile experience, symbol localization in complex 2D tactile maps becomes less efficient with age (see Fig. 3B). Finally, age was almost correlated with route performance in 2D mazes for SC (Pearson’s r = − 0.581, *p* = 0.03*, ${\mathrm{p}}_{\mathrm{bonf}}$ = 0.06, see Fig. 3C). Therefore, the older SC were, the harder cognitive map formation with 2D mazes. It is also interesting to note that, in EB only, age was positively related to performance, a result that neared significance for 2D mazes (Pearson’s r = 0.490, *p* = 0.089, ${\mathrm{p}}_{\mathrm{bonf}}$ = 0.178).

To know if better tactile explorers can build stronger cognitive maps, the relation between symbol localization time and route performance was also investigated. Correlations were only found for 3D mazes in LB (Pearson’s r = 0.604, *p* = 0.038*, ${\mathrm{p}}_{\mathrm{bonf}}$ = 0.076), and in EB (Pearson’s r = 0.622, *p* = 0.023*, ${\mathrm{p}}_{\mathrm{bonf}}$ = 0.046*). This indicates that the more efficient EB (and LB to some extent) are in exploring 3D mazes, the easier cognitive map formation becomes (see Fig. 3D). Lastly, it also appears that SBSOD scores are positively related to route performance in EB (Pearson’s r = 0.831, *p* < 0.001***) and, to some extent, LB (Pearson’s r = 0.558, *p* = 0.06), meaning that good navigators have better cognitive mapping skills in the context of our task (see Fig. 3E).

## Discussion

The goal of the present study was to investigate the impact of three-dimensional information on tactile exploration and cognitive map formation. To do so, we tested EB, LB, and SC participants in their ability to find symbols and learn the layouts of tactile 2D and 3D mazes. SC also performed the task visually. As a measure to assess cognitive map strength, participants had to recall every POI in the mazes and infer, from memory, routes between different POIs. Results showed that (1) as expected, SC immediately detected visual symbols, but took a longer time to locate these symbols when presented in the tactile modality, an effect that is most likely due to the surprisingly long time they took to detect symbols in 2D mazes (see Figs. 1 and 3A). As for the two blind groups, LB were also faster in 3D mazes, while EB did not differ between 2D and 3D mazes; (2) all three groups performed equally well in the POI recall task and were not affected by information type; and (3) SC and EB manifested stronger cognitive map formation with 3D mazes, while LB performed equally well with 2D and 3D mazes. Furthermore, SC manifested equivalent cognitive map formation between visual and tactile 3D mazes.

### Impact of 3D tactile information on exploration

Symbol localization time differences between SC, EB and LB are in line with previous studies showing that extensive tactile training and overreliance on tactile information in EB and LB can improve tactile sensitivity and, thus, the efficiency of tactile exploration^42–44^. Furthermore, it has been shown that tactile sensitivity in blind individuals, mostly EB, is less affected by aging than in SC^43,45^, which was also reflected in our study since exploring and learning 2D mazes became more arduous with age for SC, but not for EB and LB. Contrary to those in tactile 2D mazes, symbol localization times in 3D mazes were rather consistent across all three groups, indicating that enhanced tactile contrast (3D elevation of the symbols) facilitates exploration for tactilely untrained individuals and even reduces interindividual differences caused by age, blindness onset, and visual experience. This is consistent with previous studies that showed that 3D haptic perception is not affected by age^45^ and leads to equal or superior shape recognition performance in blind individuals compared to SC^42^. However, enhancing tactile contrast with 3D information also comes with significant caveats. While the advantage of using 3D information in tactile maps is the ability to change the elevation of important information, its disadvantage is that some information may be masked by protruding information and that, in the same way, hollowed information (i.e., the “descending staircase” symbol) becomes ineffective. Professionals developing 3D maps must therefore (1) avoid using hollowed information; and (2) avoid using high objects that mask less elevated information and, thus, impede exploration.

### Impact of 3D tactile information on cognitive map formation

All participants were able to infer routes with varying levels of complexity, supporting the idea that cognitive map formation is possible using visual and tactile sensory modalities^46,47^ and that blind individuals, regardless of visual experience, can form allocentric spatial representations of unfamiliar environments^46^. However, EB and SC route performance with tactile 2D mazes shed light on significant disadvantages of using tactile maps to support cognitive map formation. On the one hand, learning can be negatively impacted by tactile exploration skills and age, as shown by SC. On the other hand, learning with 2D tactile information is also limited by individuals’ prior knowledge and exposure to spatial information. This is shown by EB who experienced more challenges in forming accurate cognitive maps from 2D tactile maps compared to LB^6,24–27,48^. This difficulty may arise from EB participants’ lack of familiarity with the representational rules of visual 2D maps (i.e., floor plans and schematics)^49–53^ while prior knowledge of such representational rules (gained with visual experience) benefits less experienced tactile readers like LB^50^. Indeed, it has been shown that this performance gap is reduced when EB become more familiar with 2D images^50^ or, similarly to the present study, when using 3D shapes^42^. Additionally, 3D tactile information surprisingly led to route performance equivalent to that with vision. This finding indicates that the tactile sensory modality can carry as much relevant spatial information as vision and further emphasizes the advantage of 3D tactile information to improve cognitive map formation for all types of individuals.

### Advantages of 3D information in tactile maps

Cognitive map formation is a cognitively demanding task. This is especially true when the individual is limited to the sense of touch for which exploration is highly sequential compared to visual exploration^6,8,50^. This sequential exploration therefore is highly taxing for spatial working memory capacities and might subsequently delay or interfere with the integration of spatial information into higher-order representations. Furthermore, reading 2D maps relies on the memorization of different types of symbols that are abstract in nature and are thus more demanding on memory and attentional resources^49,54–56^. Consequently, this cognitive overload^57^ can negatively impact cognitive map formation. Since adding 3D information to tactile maps can benefit all participants regardless of age and blindness onset, we hypothesize that 3D information in tactile maps can reduce this cognitive overload by optimizing working memory capacity^57^, an advantage that resides in the following factors. First, 3D haptic perception relies less on tactile sensitivity and attention. Indeed, 2D embossed information like Braille relies mainly on one type of somatosensory receptor^58,59^ and varies according to stimuli texture^60^ and the individual’s attentional capacities^61^. On the contrary, haptic feedback from 3D tactile information involves receptor types that respond to skin stretch, muscle stretch, and articulation angle, and relies less on the individual’s ability to attend tactile stimuli at the cutaneous level^45^. 3D information is thus more advantageous for individuals who recently became blind or visually impaired or for older adults living with vision loss. As tactile sensitivity is known to decrease with age^43,45^ and as the prevalence of vision loss related to age and health conditions (e.g., diabetes) is rising, technology that is less reliant on tactile sensitivity, like 3D printed maps, would be more universally advantageous. Second, it is known that abstract symbols in traditional 2D tactile maps can be more challenging to certain people, especially individuals living with a cognitive impairment and older adults with memory deficits^62^. Compared to 2D embossed information, 3D is less reliant on abstract symbols and can display more faithful representations of objects^62^. These are thus easier to locate, identify and even memorize^39^.

Consequently, a 3D map user does not need to constantly remember the meaning of abstract symbols or pay attention to the boundaries of the navigable and non-navigable areas since routes and paths become easier to follow. 3D information can thus increase the allocation of attentional resources to the spatial learning task itself and lead to more complete cognitive maps for the same exploration time. Additionally, 3D tactile maps are less affected by interindividual differences such as tactile training and age. Thus, our research demonstrates that publicly displaying well-produced 3D maps, for example in museums, malls, or airports, would enable blind and visually impaired individuals worldwide to find their destinations more efficiently and independently in novel areas.

### Amodality of cognitive maps

It has been shown that EB, even those with no visual experience, can perform equally well to LB and SC at wayfinding tasks after extensive training^6,63–65^. However, our study shows that improving the way tactile information is presented can also improve the spatial learning process and lead to equivalent cognitive map formation without the need for extensive training. The quality of the depicted information thus seems to be a more important factor than visual or tactile experience because 3D information improves spatial learning for individuals who lack one or the other. These findings are congruent with the *amodality* hypothesis of spatial representations according to which spatial representations, including allocentric cognitive maps, are formed independently from sensory modalities and visual experience^66,67^. However, the *amodality* hypothesis remains highly debated since EB often show deficits in spatial processing^68–71^ and rely more on egocentric strategies (route knowledge) rather than allocentric ones^6,8,25,67^. Nonetheless, EB remain able to form allocentric cognitive maps and to use related wayfinding strategies for which errors often depend on how spatial information was acquired^46,72–74^. It is therefore increasingly believed that deficits arise from flawed infrastructure design that only leaves spatial information fully accessible to sighted individuals and not to those with visual impairments^66,67^. This viewpoint is supported by recent literature that shows that, when given the same amount of spatial information, EB perform as well as SC and LB in many spatial tasks and, by doing so, they activate the same cortical areas and neural networks than their sighted counterparts^66^. This emphasizes the need to improve the accessibility of spatial information and to adequately adapt and standardize the tactile maps available in public spaces^23,75^. Clearer 3D maps may give rise to more opportunities for autonomous navigation, more exposure to complex environments, and hence improved cognitive mapping skills in visually impaired populations.

### Limitations and future directions

Even though tactile maps are beneficial tools, they often provide a spatial representation from one unique aerial perspective. Indeed, to facilitate exploration and reading, blind participants often have the same viewpoint (i.e., facing North in the present study), which might lead to the formation of less flexible cognitive maps. In fact, sources of errors, especially in EB, were linked to the inability to switch between the aerial (allocentric) viewpoint and the intended egocentric viewpoint. For example, participants would confuse “left” and “right” when facing south or would use vocabulary such as “going [up or down, left or right] in the map”. Furthermore, it is known that the efficiency of tactile maps can be improved by combining their use with verbal feedback and direct exploration^46,76,77^ or, in some cases, by adding textures^54,78^. However, textures and multimodal learning were not investigated in the context of the present study, and this may have limited the spatial information that blind participants were able to absorb. Therefore, one could assume that combining 3D information with textures, verbal descriptions, and direct exploration may improve the efficiency of tactile maps.

## Conclusion

The present study investigated, for the first time with quantitative measures, the impact of information type on the ability of blind subjects to form allocentric cognitive maps and infer different routes from memory. It demonstrated that 3D information can lead to cognitive maps that are equivalent to those formed with vision by reducing the cognitive overload that stems from tactile exploration and abstract symbol memorization. Therefore, the present findings support that cognitive mapping deficits observed in early blind individuals may not arise from blindness per se, but from the inaccessibility—or inadequate adaptation—of the spatial information available in the environment. Installation of 3D printed tactile maps should be considered in public spaces to promote universal accessibility by facilitating orientation and promoting independence of blind and visually impaired travelers.

## Methods

Previous versions of this work were published as preprints^79,80^.

### Participants

The present study was approved by the Clinical Research Ethics Committee (CERC) of the University of Montreal (project # CERC-20-081-D) and was conducted in accordance with the Declaration of Helsinki. All participants gave informed consent to participate in this study. We included a total of 25 participants with complete blindness (mean age = 40.52 ± 12.34 years; 16 M and 9 F) including 11 early blind (EB, mean age = 33.45 ± 8.07 years; 9 M and 2 F) and 14 late blind (LB, mean age = 46.07 ± 12.48 years; 7 M and 7 F). Blind participants were classified according to the following criteria: if complete blindness occurred at ≤ 6 years old, participants are considered EB; if blindness onset happened after 6 years old, participants are considered LB. However, two LB (participants 12 and 13) had total blindness at 7 and 8 years old but were functionally blind since early years of life with progressive vision loss. Therefore, these participants were included in the EB group during statistical analysis, totalizing 13 participants in the EB group and 12 in the LB group. Consequently, all participants in the EB group were functionally or completely blind before the developmental period in which normally sighted children demonstrate significant improvements in spatial memory and in the use of allocentric cognitive maps and strategies^81–84^. Table 1 summarizes the characteristics of all included blind participants.

**Table 1:** Characteristics of blind participants.

#	Group	Age	Sex	Blindness cause	Blindness onset	BDI	Residual vision	SBSOD score
1	EB	50	M	ROP	~ Birth	1	–	89.52
2	EB	36	F	Glaucoma	6	0.83	LP	89.52
3	EB	30	M	Leber hereditary optic neuropathy	Birth	1	–	62.86
4	EB	31	M	Microphthalmia + glaucoma	Birth	1	LP in one eye	61.90
5	EB	29	M	ROP	~ Birth	1	–	74.29
6	EB	42	M	Retinoblastoma	3	0.93	–	83.81
7	EB	32	M	ROP	~ Birth	1	–	56.19
8	EB	33	M	Septo-optic dysplasia	Birth	1	–	79.05
9	EB	39	F	ROP	~ Birth	1	–	36.19
10	EB	25	M	Malaria	6	0.76	LP	72.38
11	EB	21	M	ROP	~ Birth	1	–	78.10
12	LB*	30	F	Glaucoma	7	0.77	–	78.10
13	LB*	43	F	Sturge-Weber syndrome	8	0.81	–	83.81
14	LB	56	F	RP	24	0.57	–	78.10
15	LB	47	M	Glaucoma	40	0.15	–	74.29
16	LB	45	F	Glaucoma + aniridia	17	0.62	–	80
17	LB	45	M	Accident	17	0.62	–	93.33
18	LB	30	M	Leber amaurosis	18	0.40	–	79.05
19	LB	56	F	RP + glaucoma	32	0.43	LP in one eye	62.86
20	LB	51	M	Accident	17	0.67	–	88.57
21	LB	57	M	Microphthalmia	20	0.65	–	74.29
22	LB	27	M	RP	17	0.37	–	56.19
23	LB	47	F	Diabetic retinopathy	22	0.53	–	74.29
24	LB	73	M	Accident	38	0.48	–	91.43
25	LB	38	F	Glaucoma	20	0.48	–	87.62

BDI, blindness duration index; EB, early blind; F, female; LB, late blind; LP, light perception; M, male; ROP, retinopathy of prematurity; RP, retinitis pigmentosa; SBSOD, Santa Barbara Sense of Direction.

*Participants who were included in the EB group according to the criteria described in the text.

We included 14 age- and sex-matched sighted controls (SC) with normal or corrected-to-normal vision (mean age = 43.43 ± 14.27 years; 8 M and 6 F). Participants had no associated neuropathy or other health conditions that could affect tactile sensitivity or mental spatial representations. All blind participants were Braille readers, fluent in French, and had previous experience with tactile maps. Before the start of the experiment, participants completed a questionnaire on their visual diagnosis, orientation and mobility (O&M) training, Braille reading efficiency, and spatial abilities, including the Santa Barbara Sense of Direction (SBSOD) scale^85,86^. To evaluate the influence of experience-dependent plasticity in both EB and LB, we also calculated the blindness duration index (BDI) according to the formula “(age–age onset blindness)/age”^87,88^. The BDI score can range from 0 to 1, indicating the proportion of life that participants have been blind, with low scores indicating recent blindness onset and high scores, longer blindness duration.

### Material

**Figure 4:**
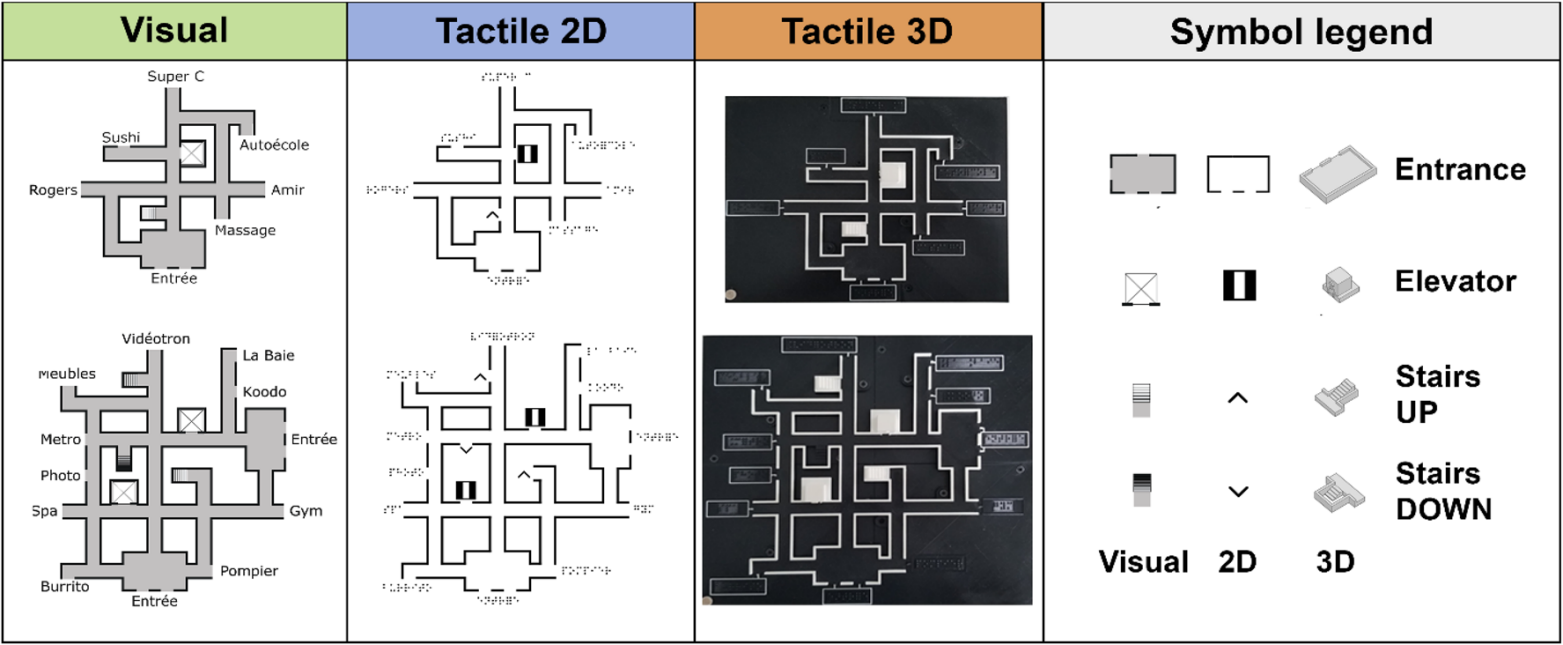
Examples of contextual mazes for every information type. The figure displays one simple and one complex maze for every information type (visual, tactile 2D and tactile 3D) and a legend of all symbols. Points of interest in tactile mazes are written in Braille. 2D, two-dimensional; 3D, three-dimensional.

**Figure 5:**
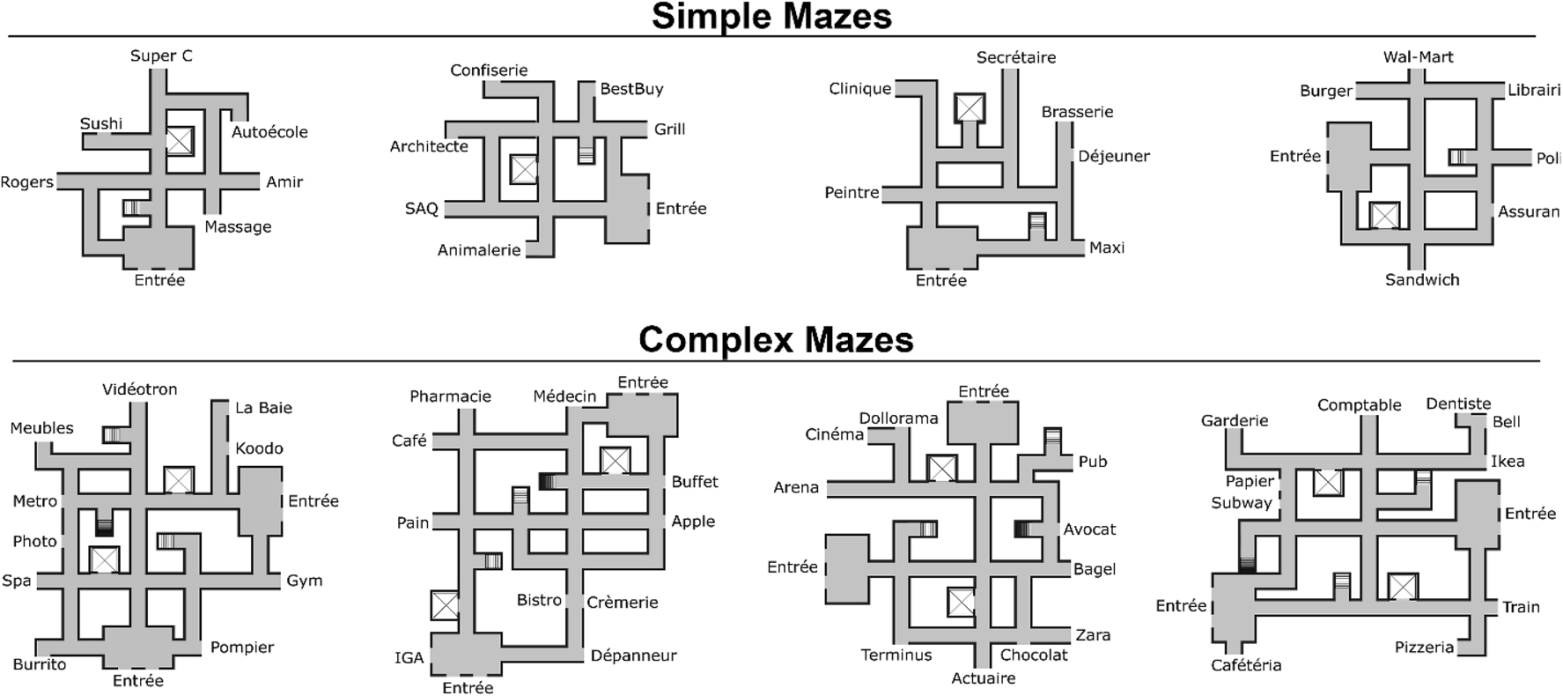
All eight mazes. The figure displays the four simple and four complex mazes in their visual format. All points of interest (POIs) were written in French and taken from a randomly generated list of words.

The research material for this study was produced with resources from the Open Science Fabrication Laboratory OptoFAB^89^. Eight different contextual mazes (C-mazes) were produced in three different information types: tactile 2D (swell paper), tactile 3D (3D printed), and visual (pictures displayed on a computer monitor). Each C-maze was composed of a network of interconnected hallways and contained entrance halls, symbols indicating floor changes (staircases and elevators), and points of interest (POIs) consisting of business names (restaurants, stores, health services, etc.) annotated in Canadian French Braille and randomly taken from a list of businesses found in the Montreal area. Furthermore, the C-mazes were also produced with two levels of complexity (simple and complex) according to the number of included features: the four simple mazes included one entrance hall, one elevator, one staircase (ascending), and six POIs; the four complex mazes had two entrance halls, two elevators, three staircases (two ascending, one descending), and ten POIs. To avoid recall bias, POIs were different for each maze. The 3D C-mazes were 3D printed with the Prusa i3 MK2S (Prusa Research, Czech Republic) printer and the 2D C-mazes were produced on traditional swell-touch paper with the PIAF Tactile Image Maker (HARPO, Poland). Figure 4 represents the C-mazes according to information type (3D vs. 2D vs. visual) and Fig. 5, according to level of complexity (simple vs. complex).

### Tasks and procedures

#### Familiarization

Before the experiment, participants were familiarized with the mazes and their symbols by consulting legends of all three information types. During this period, participants were given verbal descriptions of the symbols with the goal of being subsequently able to locate and identify them without the help of the experimenter. Then, participants were introduced to the *contextual maze learning paradigm* and its three phases (see “Contextual maze learning paradigm” section) using a training C-maze (supplementary file 1) with reduced content (one entrance, one elevator, one ascending staircase, one descending staircase, and 3 POIs) produced in 2D, 3D and visual mediums. Participants were encouraged to explore the entirety of the mazes and to locate the different symbols and POIs. Then, participants were asked to elaborate routes between different POIs while being able to consult the mazes with their hands or eyes. If participants made errors or used vocabulary that the experimenter did not understand, the experimenter would further discuss with the participant to ensure (1) the participant understood the task; and (2) the experimenter understood the participant’s answers and vocabulary. Once participants understood and performed well in two subsequent routes, the experiment could begin. The familiarization protocol (legend and training c-maze in all three mediums) lasted approximately 15 min in total for each participant.

#### Experiment design

Each participant completed 8 blocks of the C-maze learning paradigm, one for each C-maze (Fig. 5). The order and information types in which C-mazes were presented were randomly assigned for each participant in a way that respected the following system:

*EB and LB groups*. Blind participants were given four 3D (two simple, two complex) and four 2D (two simple, two complex) C-mazes across two experimental sessions. Blocks always alternated between 2D and 3D mazes. Participants were randomly distributed into two categories: starting with either a 2D or 3D maze. Following this system, the first session began with two simple C-maze blocks and then two complex C-maze blocks, while the second session began with two complex C-maze blocks and ended with two simple C-maze blocks (AB-BA order).

*SC group*. SC participants were given four visual C-mazes (two simple, two complex) as visual controls and four tactile C-mazes including two 3D (one simple, one complex) and two 2D (one simple, one complex) mazes across two experimental sessions. In the first session, participants performed four blocks of visual C-mazes in the following order: two simple C-maze blocks followed by two complex C-maze blocks. In the second session, blocks alternated between 2D and 3D mazes, and participants were randomly distributed into two categories: starting with a 2D or a 3D maze. Following this system, the session started with two simple C-maze blocks and ended with two complex C-maze blocks. For tactile C-mazes, SC participants were blindfolded and only saw the mazes after completing the experiment.

#### Contextual maze learning paradigm

**Figure 6:**
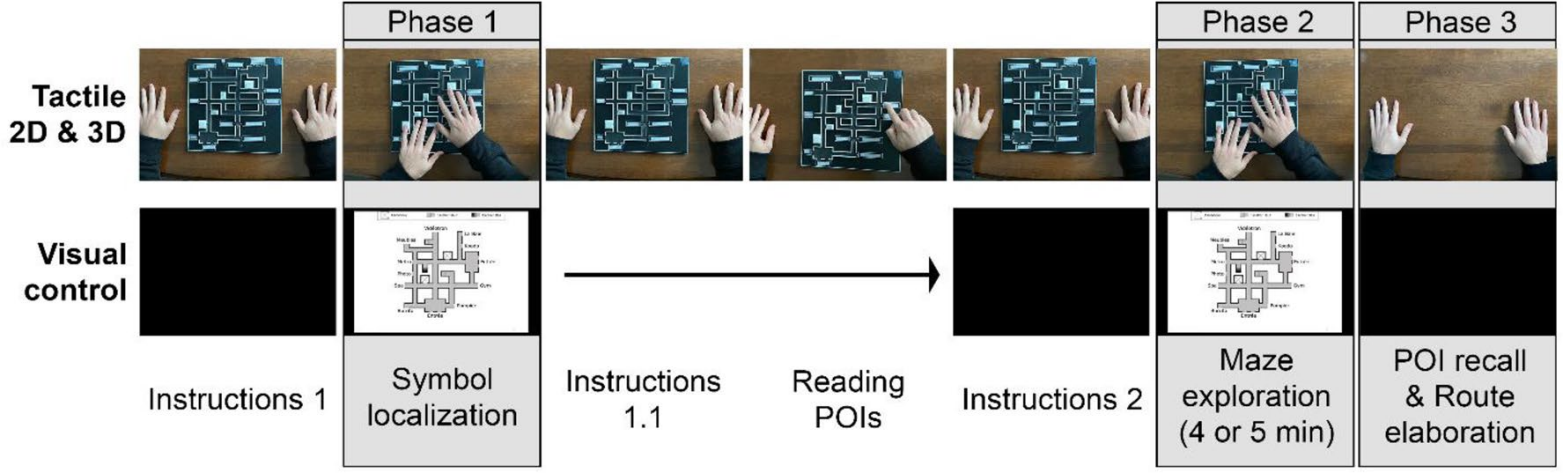
Contextual maze learning paradigm and its sequence represented from left to right. One block of the paradigm included three phases divided by instruction periods in which touching or seeing (monitor turned off) the maze was impossible for participants. During instructions 1, participants were explained phase 1 (symbol localization). In phase 1, symbol localization time (time to detect and identify symbols) was recorded. During instructions 2, participants were explained phase 2 (learning the maze as much as possible in the given time). In phase 2, participants had 4 or 5 min (according to the maze complexity level) to explore and learn the maze. Finally, in phase 3, participants could no longer explore the maze and were asked questions about the maze content (POI recall and route elaboration). The tactile conditions included one extra step: instructions 1.1. explaining the POI reading task and the POI reading task, in which POIs were read aloud by the participant (EB and LB who read Braille) or by the experimenter guiding the participant’s hand (for SC who do not read Braille). POIs, points of interest; 2D, two-dimensional; 3D, three-dimensional.

All steps of the experiment were preceded by a period of instructions during which the experimenter explained the task and the participant could not see or touch the maze. A complete diagram of the paradigm can be found in Fig. 6.

*Phase 1: Symbol localization (detection* + *identification)*. The C-maze was either placed on the table in front of the participant (tactile 2D or 3D condition) or displayed on a monitor (visual condition). Participants were asked to explore the maze either with their hands (tactile conditions) or with their eyes (visual condition) with the goal of detecting and identifying all symbols (entrances, elevators, and staircases) present in the maze. Symbol localization (detection + identification) times and order of localization were collected during that stage. In tactile conditions, the experimenter then made sure that the participants could read all POIs written in Braille. To do so, blind participants were asked to browse the perimeter of the maze and to read POIs aloud, whereas SC participants had their hand guided by the experimenter who verbally identified POIs for them.

*Phase 2: Maze exploration and learning*. This stage consisted of a maze memorization task. During a limited period of either four minutes (simple mazes) or five minutes (complex mazes), participants were asked to freely explore the given maze with the goal of learning its layout as much as possible. For tactile 2D and 3D conditions, maze exploration could be done with both hands. As SC participants could not read Braille, the experimenter verbally identified POIs every time the participants touched one. Aside from this interaction, the experimenter did not make any interventions. Once the time was up, the tactile maze was removed from the participants’ reach or the monitor displaying the visual maze was turned off.

*Phase 3: POI recall and route elaboration*. The goal of phase 3 was to test the cognitive map formed through exploration in phase 2. Participants were first asked to recall all POIs that were present in the given maze. If some POIs were forgotten, the experimenter reminded their names to the participant. Then, the route elaboration task consisted of six questions that necessitated participants to mentally represent routes related to the map elements and verbalize them. These questions were of two types: (1) route completion: the experimenter gave a route and participants were tasked to identify the POI at which the route ended; (2) route elaboration: participants were asked to elaborate a route between two POIs. To complete the route elaboration task, participants were told to roleplay and give their answers like they would if guiding someone who needed help reaching their destination in the maze.

#### Performance scoring

Since route elaboration is a cognitively complex task and participants were allowed to use their own strategies and vocabulary, responses were prone to various sources of errors. To circumvent this limitation, each answer was scored by four independent reviewers: three trained orientation and mobility (O&M) specialists (including authors M.B., C.V.A., & J.P.N.) who were either present during the experimentation or were provided audio recordings, and who have the experience to subjectively evaluate routes according to their experience with the clientele; and one research assistant provided with anonymized audio recordings and a strict grading system to objectively grade each answer. The final score for each answer was the average of the four independent scores. More detailed information regarding the performance scoring protocol can be found in supplementary file 2.

Since a double-blind experiment was not achievable due to the nature of the tactile stimuli, the study could be subject to confirmation bias from participants and experimenters. To circumvent this limitation, all questions were formulated in neutral language that did not advantage any of the information types. Moreover, we ensured that at least two O&M specialists and the research assistant—tasked with the objective evaluation—were: (1) absent during data collection; and (2) provided with anonymized audio recordings containing no information with which they could identify the participant’s group or the information type.

### Statistical analysis

The collected data consisted of the three following dependent variables: symbol localization times (in seconds, it includes the time taken to detect and identify symbols), and performance (in %) in the POI recall and route elaboration tasks. Statistical analyses of the collected data were performed using JASP version 0.14.1, an open-source graphical program developed by the University of Amsterdam^90^. The effects of factors such as group (between subject, three levels: SC vs. EB vs. LB), type of information (within subject, three levels: visual mazes vs. tactile 2D mazes vs. tactile 3D mazes), complexity (within subject, two levels: simple vs. complex mazes), and symbol identity (within subject, four levels: entrance vs. elevator vs. ascending staircase vs. descending staircase) on the dependent variables were analyzed with mixed factor ANCOVAs with age and sex entered as covariates. Greenhouse–Geisser correction to the degrees of freedom were applied on analyses where the assumption of sphericity of data was violated as detected by significant Mauchly’s tests. Normality of the data was visually checked with Q-Q plots, and a generalized linear mixed model (GLMM) analysis was performed when deviations from normality were observed. We also reported the effect of covariates (age and sex) only when they had a significant effect on the dependent variables or interacted with other factors of interest.

We first analyzed data from the SC group independently. Symbol localization times were analyzed using a 3 (information type) × 2 (complexity) ANCOVA and a 3 (information type) × 4 (symbol identity) ANCOVA, while performance in POI recall and route elaboration was analyzed using 3 (information type) × 2 (complexity) ANCOVAs. Then, we performed analyses to test how the three groups compared to each other in tactile 2D and 3D conditions and how they were affected by the different factors. Symbol localization times were analyzed using a 3 (group) × 2 (information type) × 2 (complexity) ANCOVA and 3 (group) × 2 (information type) × 4 (symbol identity) ANCOVA, while performance in POI recall and route elaboration was analyzed using 3 (group) × 3 (information type) × 2 (complexity) ANCOVAs. Simple main effects tests and post-hoc T-tests were then performed to locate and identify significant differences in localization times and performance. Lastly, we investigated if there were correlations between age, blindness onset, BDI, SBSOD scores, and the different dependent variables using Pearson’s R and Spearman Rho tests. For every test, the significance level of α = 0.05 was chosen in order to accept or reject the null hypothesis (no effect, no differences or no correlation).

## Supplementary Information

Supplementary Information.

## Data Availability

The data and all materials for the experiments reported here are available. Access to the data can be requested by contacting the corresponding author.
