# Supplementary material for: Cognitive map formation in the blind is enhanced by three-dimensional tactile information
Source: Sci Rep. 2023 Jun 15;13:9736. doi: 10.1038/s41598-023-36578-3 (PMC10272191; doi:10.1038/s41598-023-36578-3)
Supplement: Supplementary file 1 — Supplementary Information. [file 41598_2023_36578_MOESM1_ESM.pdf]

Supplementary Material

1. Instruction given to the participant

"Imagine yourself inside the building represented in the tactile map, standing at the starting point. Now imagine that someone comes to you and asks you how he/she can get to a specific location (destination). Your job is to explain them the route in a way that if this person follows your instructions, he/she can reach this specific location without making any errors and/or getting lost. Please add as much information as possible."

2. Instruction given to orientation and mobility specialists

As an orientation and mobility specialist, you can evaluate the abilities of our participants to reach destinations in an environment learned with a tactile map. Please rate the routes by judging vocabulary and strategies used by blind participants to reflect the individual's functioning.

Rating system in %. Here are some guidelines, but you can use intermediate gradation.

- 0%: the route is false, incomplete, or not even attempted.
- 25%: the participant is going in the good direction but does not complete half of the route.
- 50%: half of the route is completed successfully, but the participant does not reach the destination
- 75%: the route is generally good, but missing information leaves possibilities for someone to make errors and get lost if the instructions are followed.
- 100%: the route is complete and contains all necessary information and instructions to reach the destination without any possibilities to make errors.

3. Instruction given to the research assistant

Please listen to the audio recording containing the answers given by the participant. On the map, please draw the route you are listening to. If the participant does not reach the destination, please draw the "good route" (the route the participant should have given to have a 100% score) from the starting point to the destination. Then count the score for this "good route", then the score for the route attempted by the participant. To score this "attempted route", sum up steps that overlap with the "good route" and subtract steps (1 tile walked = -1 point) taken from the error point (the last point that overlaps with the good route). When the participant reaches the destination but does not say all information (ex: "turn right" when the good route is "turn right at the third intersection"), all steps in the "error-prone" zone have half values. In this example, the "error-prone" zone is between the first intersection where it is possible to turn right and the third intersection.

Tile values:

- Tile = 1 point
- Tile with decision point (2 possible paths) = 2 points
- Tile with decision point (3 possible paths) = 3 points

4. Contextual maze and route elaboration protocol

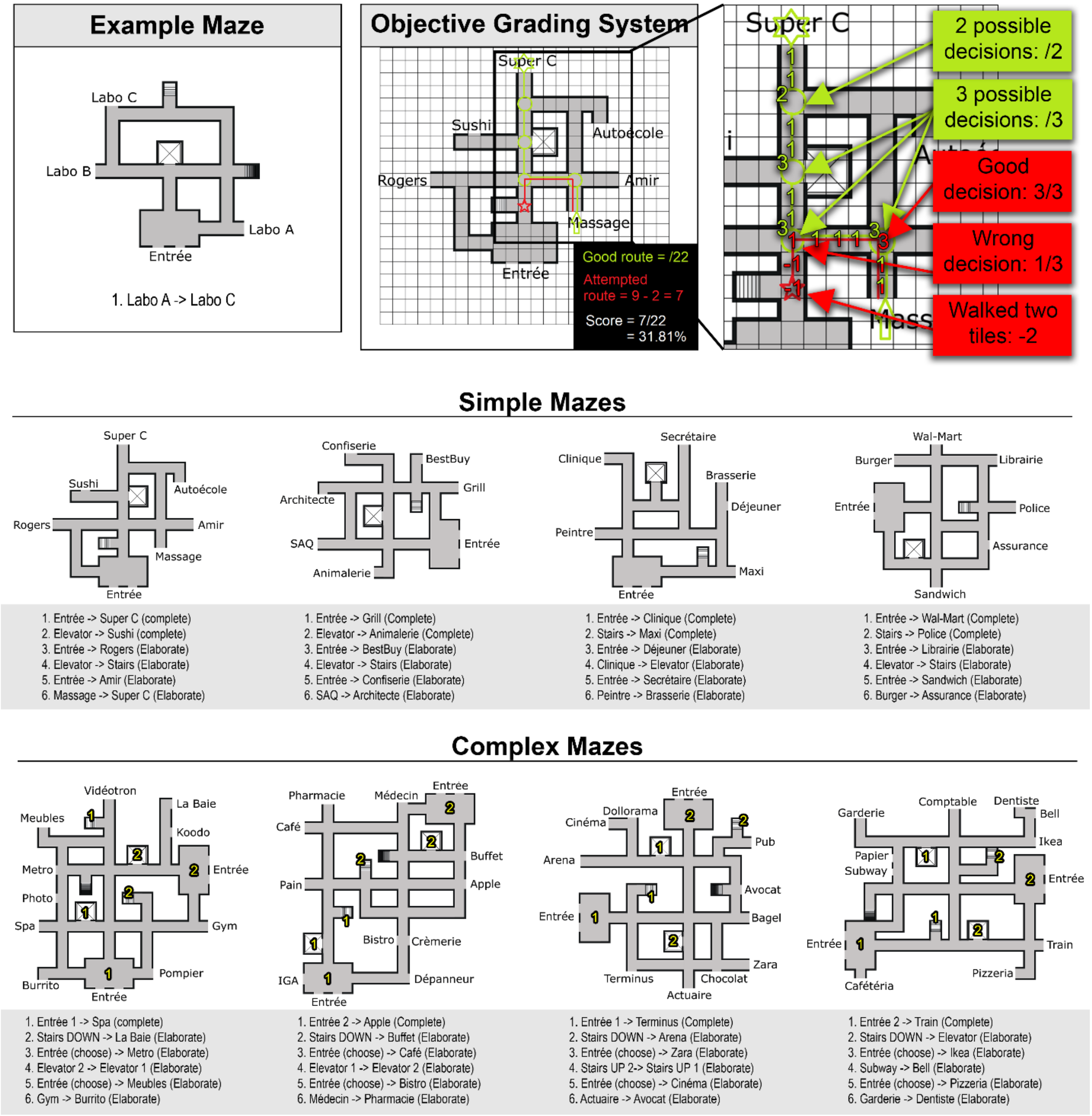

Supplementary figure 1. The contextual maze and route elaboration protocol. On the upper left is displayed the example maze used during familiarization. On the upper right, an example of the objective grading system used by the research assistant, comparing the "good route" (in green) and the route attempted by the participant (in red). The rest of the figure displays all the mazes and the route elaboration questions. Questions marked as "complete" were routes given by the experimenter, the participant had to name the ending point of the route. Questions marked as "elaborate" are route that needed to be elaborated in entirety by the participant who was given the starting point and destination.

5. Inter-rater agreement

We also verified the agreement between the four raters (three trained orientation and mobility specialists and the research assistant using the objective grading system) for all scores (6 routes x 8 C-mazes x 39 participants = 1872 rating scores / rater). Since the rating scores is a continuous variable between 0 and 100, we first evaluated the inter-rater reliability. First, we measured the intraclass correlation between the three specialists, a test that revealed that their given scores were highly correlated ($ICC_{1,1} = 0.966$, 95% CI [9.63, 9.68]). Then, we evaluated the pairwise correlation between the average scores from the three specialists and the objective rating scores performed by the research assistants with a Pearson's r test, this test revealed that the subjective and objective scores were also highly correlated (Pearson's $r = 0.927$, $p<0.001$***, 95% CI [9.20, 9.33]).
